# *Scutellaria barbata* Inhibits Hepatocellular Carcinoma Tumorigenicity by Inducing Ferroptosis of Hepatocellular Carcinoma Cells

**DOI:** 10.3389/fonc.2022.693395

**Published:** 2022-03-07

**Authors:** Yue Li, Jiongshan Zhang, Kun Zhang, Yan Chen, Wei Wang, Hongjie Chen, Zengcheng Zou, Yongwei Li, Min Dai

**Affiliations:** Department of Traditional Chinese Medicine, The Third Affiliated Hospital, Sun Yat-Sen University, Guangzhou, China

**Keywords:** hepatocellular carcinoma, traditional chinese medicine, *scutellaria barbata*, ferroptosis, lipid peroxidation

## Abstract

Ferroptosis is caused by accumulation of iron-dependent lipid peroxidation, which is characterized by reduction in cell volume and increase in mitochondrial membrane density. Studies have shown that ferroptosis contributes to the development and progression of numerous major diseases, including hepatocellular carcinoma (HCC). As a unique biomedical resource, Traditional Chinese Medicine (TCM) has been widely used in the treatment of HCC. In this present study, *Scutellaria barbata* was used to treat HCC cells *in vitro*, and the results revealed that *S. barbata* suppressed HCC cell growth through inducing ferroptosis. Next, the exploration of the molecular mechanism on how *S. barbata* induced ferroptosis in HCC cells suggested that *S. barbata* may induce ferroptosis by promoting iron perioxidation and lipid ROS metabolism. Finally, *S. barbata* also inhibited HCC tumorigenicity *in vivo* by inducing ferroptosis of HCC cells. These results provided theoretical basis for explaining the mechanism of TCM treatment for HCC and offered therapeutic opportunities for HCC patients.

## Introduction

Despite the progress in diagnosis and treatment, hepatocellular carcinoma (HCC) is still the sixth most common malignancy and the third principal cause of cancer-related mortality worldwide (1). HCC is the result of chronic liver disease and associated with nonalcoholic steatohepatitis, alcoholic hepatitis, viral hepatitis, and cirrhosis (2). Due to the high incidence of hepatitis B virus (HBV) infection, HCC poses a serious threat to the health of the population in China and Africa, especially in the sub-Saharan region (3, 4). Methods for the early diagnosis of HCC is limited, and hence, most people lose the opportunity of surgical therapy. Less than 18% of HCC patients remain to have a dismal 5-year overall survival (5). Due to the difficulties in the early diagnosis of HCC and poor prognosis for HCC patients, it is urgently needed to discover novel targets and develop novel therapies for HCC treatment.

Recently, increasing data shows Traditional Chinese Medicine (TCM) play an active role in the treatment HCC (6). TCMs have long been utilized in disease prevention, including HCC. Accumulated Chinese herbal compounds have been isolated and show preventive effects on the occurrence of HCC, such as *Scutellaria barbata*, ellagitannin, ardipusilloside-I, *Annona squamosa* seeds, *Panax*, and gypenoside (7–13). TCMs play an important role in ameliorating clinical symptoms, improving quality of life, preventing recurrence and metastasis, suppressing tumor progression, and prolonging survival period of HCC patients (14). As a critical medical resource for the development of novel treatments for HCC, TCM compound can cause HCC proliferation inhibition, cell apoptosis, cell cycle arrest, autophagy, cell aging (13, 15–18).

As a new identified type of cell death, ferroptosis is probably induced by TCM. Ferroptosis is caused by accumulation of iron-dependent lipid peroxidation, and it is involved in many major diseases including neurodegenerative diseases, ischemia–reperfusion injury, and a series of cancers (19–27). Ferroptosis is different from other major forms of regulated cell death (RCD) including apoptosis. It is an iron-dependent cell death with the accumulation of lipid peroxidation and regulated by a unique set of genes, such as iron-responsive element-binding protein 2 (IREB2), ATP synthase F0 complex subunit C3 (ATP5G3), citrate synthase (CS), acyl-CoA synthetase long chain family member 4 (ACSL4), solute carrier family 7 (SLC7A11), and glutathione peroxidase 4 (GPX4) (28). SLC7A11 is a member of the cystine-glutamate antiporter; the inhibition of SLC7A11 leads to the accumulation of reactive oxygen species (ROS) and subsequent ferroptosis (28). As a selenoprotein, the catalytic site of GPX4 protein contains a key selenocysteine residue, which can partially protect HCC cells from ferroptosis (29). However, the role of TCM in ferroptosis in HCC cells remains unclear. In this present study, we committed to screen the target TCM for inducing ferroptosis in HCC. In addition, we elucidated the molecular mechanism of *S. barbata* to induce ferroptosis in HCC, which may provide new treatments and targets for HCC.

## Materials and Methods

### Reagent

*Scutellaria barbata* was obtained from The Third Affiliated Hospital of Sun Yat-sen University. Roswell Park Memorial Institute (RPMI)-1640 (SH30022.01B), fetal bovine serum (FBS) (SH30087.01), penicillin (SH30010), and phosphate-buffered saline (PBS) (SH30256.01B) were brought from GE™ Hyclone company (Logan, UT, USA). Lactate dehydrogenase (LDH) Cytotoxicity Colorimetric Assay kit (K311-400) was brought from BioVision Company (Milpitas, CA, USA). Iron ion colorimetric detection kit (E1042) was brought from Beijing Prilie Gene Technology Co. Ltd (Beijing, China). JC-1 kit (T-3168) and BODIPY™ 581/591 C11 probe (D3861) were brought from Life Technologies Corporation (Carlsbad, CA, USA). PrimeScript II 1st Strand cDNA Synthesis Kit (D6210A) and SYBR Premix ExTaq II were brought from TaKaRa Company (Dalian, Liaoning, China). TRE-Trizol was brought from Invitrogen Company (Carlsbad, CA, USA). EdU (LM067) was brought from LMAIBio Company (Shanghai, China).

### Cell Culture

SMMC-7721, HepG2, and Huh7 cells were maintained with Dulbecco’s modified Eagle’s medium (DMEM) (SH30022.01B, Hyclone) containing 10% FBS (SH30087.01, Hyclone), 100 U/ml penicillin (SH30010, Hyclone), and 100 mg/ml streptomycin in a humidified atmosphere at 37°C with 5% CO_2_.

### Drug IC50 Test

SMMC-7721 cells (1 × 10^4^) (or HepG2, Huh7) were plated in a 96-well plate. Cells were cultured with RPMI-1640 containing 10% FBS and 0, 3.15, 6.3, 12.5, 25, or 50 mg/ml of *S. barbata* for 48 h. Inhibitory rate of cell proliferation was then identified by Cell Counting 8 Kit (CCK8; Beyotime, Shanghai, China) following the manufacturer’s protocol. Inhibitory rate = (1 − mean OD value of experimental group/mean OD value of control group) ×100%. Next, the IC50 of the drug was calculated by the inhibition rate.

### Lactate Dehydrogenase and Cytotoxicity Test

Cells (adherent or suspended cells) were collected and washed with 1× medium (e.g., 1% serum or BSA) and subsequently seeded in 96-well plates, and blank control wells were added 200 µl medium to three repeating microporous plates. All other holes must be subtracted from the blank control value. A total of 2 × 10^4^ cells/well were cultured in 200 µl of assay medium with three repeated micropores in low control wells, while 1–2 × 10^4^ cells/well were cultured in a 200-µl assay medium containing 1% Triton X-100 with three repeated micropores in high control wells. In addition, 1–2 × 10^4^ cells/well were cultured in 200 µl of assay medium with three repeated micropores in sample wells. The cells were incubated for 20 min in an incubator of 5% CO_2_, 90% humidity, and 37°C. Then, the cells were centrifuged with a centrifugal force of 250*g* for 10 min. Suspended cells were added into the 96-hole plate. Next, 100 µl/well of reaction solution was added and incubated at room temperature (RT) in the dark for 30 min. The absorbance at wavelength of 490–500 nm of samples were measured, and the cell cytotoxicity was identified by the following formula: (OD of sample − OD of low control)/(OD of high control − OD of low control) × 100%.

### Iron Ion Concentration Detection

For cells, drugs were dissolved in PBS and diluted with complete medium into their IC50 concentration. A total of 1 × 10^5^ cells/well were seeded in 24-well plates and treated with *S. barbata* for 48 h. Iron ion concentration was then determined using iron ion colorimetric detection kit following the manufacturer’s protocol.

For tumor tissues, the tumor samples were washed with 2 ml of cold normal saline for two times, and then, the normal saline was suck up (if it is not tested immediately, it can be frozen at −20°C). Normal saline (200 μl/g) was added into tumor samples to prepare tissue homogenate, and tissue homogenate was centrifugated to the collected supernatant for iron ion colorimetric detection.

### Mitochondrial Membrane Potential Detection

*Scutellaria barbata* was dissolved in PBS and diluted with complete medium into its IC50 concentration. A total of 1×10^6^ cells/well were seeded in six-well plates and treated with *S. barbata* for 4 h. Mitochondrial membrane potential (MMP) was then determined using JC-1 Kit (Life Technologies Corporation, Jersey City, NJ, USA) following the manufacturer’s protocol. Red fluorescence indicates high MMP, while green fluorescence represents the dissipation of MMP. The ratio of red to green fluorescence intensity demonstrates the total/monomer ratio of JC-1.

### Reactive Oxygen Species Assay (C11-BODIPY Probe)

*Scutellaria barbata* was dissolved in PBS and diluted with complete medium into its IC50 concentration. A total of 1–2 × 10^6^ cells/well were seeded in six-well plates and treated with *S. barbata* for 48 h. ROS was then determined using C11-BODIPY probe following the manufacturer’s protocol. The ratio of emission fluorescence intensity at 590 to 510 nm gave a reading of ROS in the cell.

### Realtime Polymerase Chain Reaction

After the treatment of *S. barbata*, real-time PCR was performed to verify the influence of *S. barbata* on GPX4, SLC7A11, IREB2, and ACSL4 in SMMC-7721 (or HepG2, Huh7) cells or in *in vivo* tumors. Total RNA was isolated from cells or tumor tissues using the Trizol reagent (Invitrogen) and then reversed transcribed by the PrimeScript II 1st Strand cDNA Synthesis Kit (TaKaRa, Dalian, Liaoning, China). Subsequently, real-Time PCR was performed by the StepOnePlus system (Applied Biosystem, Foster City, CA, USA) using TaKaRa SYBR Premix ExTaq II. Primer sequences are shown in **Table 1**.

**Table 1:** Primers used in this study.

Primer name	Primer sequence (5’–3’)	Product (bp)
GPX4-F	TCAGCAAGATCTGCGTGAAC	217
GPX4-R	GGGGCAGGTCCTTCTCTATC	
IREB2-F	GCACCGGATTCAGTTTTGTT	201
IREB2-R	CTTAGCGGCAGCACTATTCC	
ACSL4-F	AATGCAGCCAAATGGAAAAG	152
ACSL4-R	CACAGAAGATGGCAATGGTG	
SLC7A11-F	GGCAGTGACCTTTTCTGAGC	224
SLC7A11-R	TCATTGTCAAAGGGTGCAAA	
GAPDH-F	AACGGATTTGGTCGTATTGGG	207
GAPDH-R	CCTGGAAGATGGTGATGGGAT	

### Western Blot Analysis

To detect the cellular level of target proteins, protein extracted from cells or tumor tissues were detected by Western blotting. Protein concentrations were determined by BCA Protein Assay Kit (BioRad, Hercules, CA, USA). Next, 30–40 μg of soluble proteins of each sample was separated using sodium dodecyl sulfate (SDS)-polyacrylamide gel electrophoresis and electrophoretically transferred onto polyvinylidene difluoride (PVDF) membranes (Millipore, Billerica, MA, USA). The primary antibodies used in the present study were diluted into 5% nonfat milk as follow: GPX4 antibody (PAC994Hu01, USCN, Wuhan, China, 1:1,000), ACSL4 antibody (A04372-2, Boster, Wuhan, China, 1:1,000), CCRB1 (SLC7A11) antibody (bs-6883R, Bioss, Beijing, China, 1:1,000), IREB2 antibody (PAH789Hu01, USCN, 1:1,000), and GAPDH antibody (KC-5G5, Aksomics, Shanghai, China, 1:10,000). After blocking by 5% nonfat milk for 1 h at RT, membranes were incubated with primary antibodies at 4°C overnight. GAPDH was used as the internal reference. Next, membranes were washed twice by Tris-buffered saline (TBS) with 0.1% Triton and incubated with horseradish peroxidase (HRP)-conjugated secondary antibody (4050-05, Southern Biotech, 1:20,000) for 1 h at RT. Finally, Clarity™ Western ECL substrate (Bio-Rad, Hercules, CA, USA) was utilized to visualize the protein bands, and quantification analysis was performed by using ImageJ software.

### EdU Assay

For EdU detection, first, each group of cells was pre-cultured with diluted EdU medium for 3 h according to the instructions. Then, cells were washed twice in PBS, then fixed in 4% paraformaldehyde for 30 min, infiltrated with 0.1% Triton X-100 for 10 min, washed with PBS, added with 100 μl Apollo staining solution to each well cells, incubated in the dark for 30 min, and then stained the nucleus. 4′,6-Diamidino-2-phenylindole (DAPI) solution was applied, and then the results were visualized by a fluorescence microscope.

### *In Vivo* Xenograft Model

Five-weeks-old male BALB/c nude mice used in this study were purchased from Beijing Vital River Laboratory Animal Technology Co., Ltd (Beijing, China), and the housing conditions were as follows: a 12-h light/dark cycle, normal grade, and free to eat and drink. For the xenograft study, 1 × 10^7^ cells in 100 µl PBS were inoculated subcutaneously into nude mice. The nude mice were randomly divided into two groups (intragastric administration of 140 g/10 g, *S. barbata* or PBS) with five mice in each group. The groups were labeled as administration group or unadministration group. Subcutaneous injection and administration were conducted according to the above groups. Cervical dislocation was performed in nude mice, and the tumor size and weight were measured. The tumor was collected for subsequent experimental detection. All animal experimental procedures were performed strictly in accordance with the Ethic Committee of The Third Affiliated Hospital of Sun Yat-Sen University.

### Statistical Analysis

All experiments in this study were repeated at least two times, and average values of three experiments were presented as the mean standard deviation (SD) calculated by STDEV formula in Excel. The significance of all data was estimated by Tukey’s multiple-comparison test in the ANOVA analysis by the Sigma Stat 3.5 software. Importantly, statistical significance was accepted when *p* < 0.05.

## Results

### *Scutellaria barbata* Suppresses HCC Cell Growth

**Figure 1:**
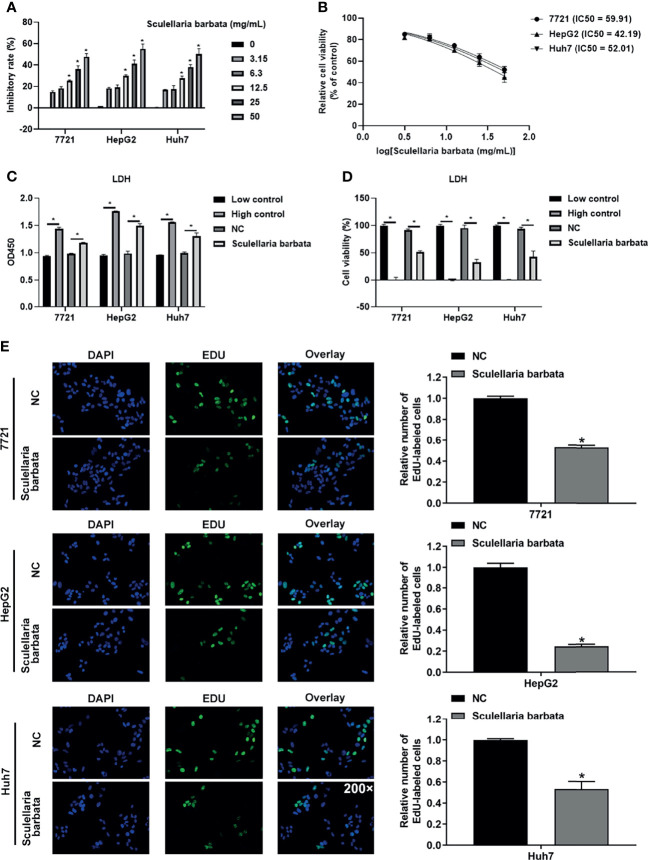
*Scutellaria barbata* suppresses HCC cells growth. **(A)** Inhibitory rate of HCC cell lines (SMMC-7721, HepG2, and Huh7) under different concentrations of *S. barbata* treatment by CCK8 assay. **(B)** IC50 value of *S. barbata* on SMMC-7721, HepG2, and Huh7 cells. **(C)** LDH level was detected by ELISA in HCC cell lines (SMMC-7721, HepG2, and Huh7) after *S. barbata* treatment. **(D)** Cytotoxicity of *S. barbata* on SMMC-7721, HepG2, and Huh7 cells. **(E)** EdU assay of *S. barbata* on SMMC-7721, HepG2, and Huh7 cells. Data were representative of three independent experiments and analyzed by unpaired t-test. Error bars denote SD. Magnification: 200×. *p < 0.05.

To evaluate the effect of *S. barbata* on HCC cell lines (including SMMC-7721, HepG2, and Huh7), CCK8 assay was performed to detect the growth of HCC cells after the *S. barbata* treatment. Results showed that *S. barbata* significantly inhibited the growth of HCC cells in a dose-dependent manner, and the inhibitory rate was enhanced by increasing *S. barbata* dosage (**Figure 1A**). Moreover, the IC50 concentration of *S. barbata* was 44.26 mg/ml (SMMC-7721), 42.19 mg/ml (HepG2), and 52.01 mg/ml (Huh7), respectively, which was used for the subsequent experiments (**Figure 1B**). Then, the marker of cell-death-released LDH was detected by ELISA. Results indicated that treatment of *S. barbata* dramatically increased the level of released LDH in HCC cells (**Figure 1C**). The cytotoxicity was significantly increased in HCC cells treated with *S. barbata* (**Figure 1D**). In addition, EdU staining assay was performed to detect the cell proliferation and revealed that the treatment of *S. barbata* significantly suppressed the growth of HCC cells (**Figure 1E**). All these data suggested that *S. barbata* may suppress HCC cell growth through inducing HCC cell death.

### *Scutellaria barbata* Induces Ferroptosis in HCC Cells

**Figure 2:**
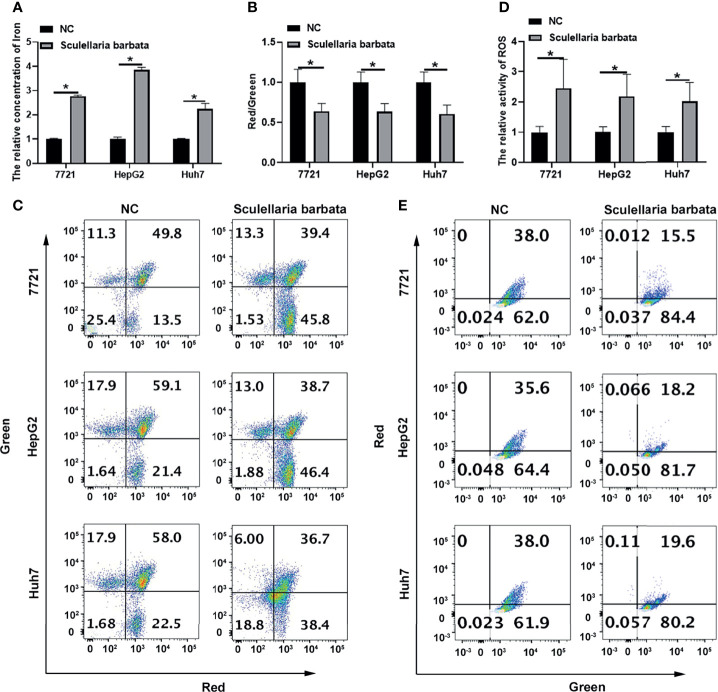
*Scutellaria barbata* induces ferroptosis in HCC cells. **(A)** Iron concentration in *S. barbata*-treated HCC cell lines (SMMC-7721, HepG2, and Huh7) was detected by ELISA. **(B, C)** The mitochondrial membrane potential of *S. barbata*-treated HCC cell lines (SMMC-7721, HepG2, and Huh7) was detected by JC-1 kit through flow cytometry. **(D, E)** The ROS level of *S. barbata*-treated HCC cell lines (SMMC-7721, HepG2, and Huh7) was detected by C11-BODIPY probe on flow cytometry. Data were representative of three independent experiments and analyzed by unpaired t-test. Error bars denote SD. *p < 0.05.

Ferroptosis is caused by the accumulation of iron-dependent lipid peroxidation, which is characterized by reduction in cell volume and increase in mitochondrial membrane density. Results showed that *S. barbata* significantly increased the iron concentration in HCC cell lines, including SMMC-7721, HepG2, and Huh7 cells (**Figure 2A**). Moreover, mitochondrial membrane potential identified by JC-1 staining was decreased in HCC cells after the *S. barbata* treatment (**Figures 2B, C**). Furthermore, the level of ROS detected by C11-BODIPY probe on flow cytometry was dramatically reduced in HCC cells treated with *S. barbata* (**Figures 2D, E**). Taken together, these results suggested that *S. barbata* induced ferroptosis in HCC cells.

### *Scutellaria barbata* Induces Ferroptosis Through Regulating Genes Involved in Lipid ROS Metabolism and Iron Perioxidation in HCC Cells

**Figure 3:**
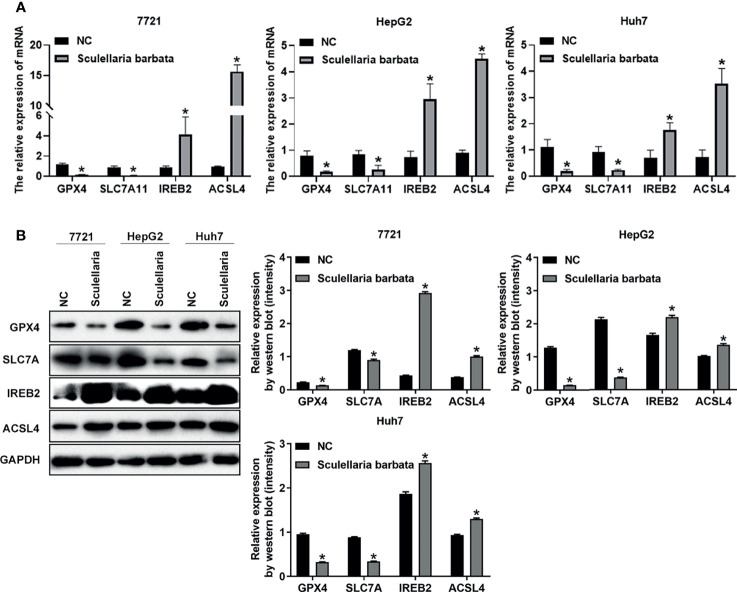
*Scutellaria barbata* induces ferroptosis through regulating genes involved in lipid ROS metabolism and iron perioxidation in HCC cells. **(A)** The mRNA levels of GPX4, SLC7A11, IREB2, and ACSL4 were detected by RT-PCR in HCC cell lines (SMMC-7721, HepG2, and Huh7) with or without *S. barbata* treatment. **(B)** The protein levels of GPX4, SLC7A11, IREB2, and ACSL4 were detected by Western blot analysis in HCC cell lines (SMMC-7721, HepG2, and Huh7) with or without *S. barbata* treatment. Data were representative of three independent experiments and analyzed by unpaired t-test. Error bars denote SD. *p < 0.05.

Next, the molecular mechanism of *S. barbata* inducing ferroptosis in HCC cells was explored. Lipid ROS metabolism-related genes GPX4 and SLC7A11 were ferroptosis inducer (30, 31), whereas iron perioxidation-related gene IREB2 and CoA metabolism-related gene ACSL4 were ferroptosis inhibitor (32, 33). The mRNA and protein levels of these four genes were detected in HCC cell lines (SMMC-7721, HepG2, and Huh7), respectively. Results revealed that both mRNA and protein levels of GPX4 and SLC7A11 were reduced after *S. barbata* treatment in HCC cells (**Figures 3A, B** **)**. By contrast, both mRNA and protein levels of IREB2 and ACSL4 were elevated after *S. barbata* treatment (**Figures 3A, B** **)**. These data suggested that *S. barbata* may induce ferroptosis through regulating the expression of genes involved in lipid ROS metabolism and iron perioxidation in HCC cells.

### *Scutellaria barbata* Inhibits HCC Tumorigenicity *In Vivo*

**Figure 4:**
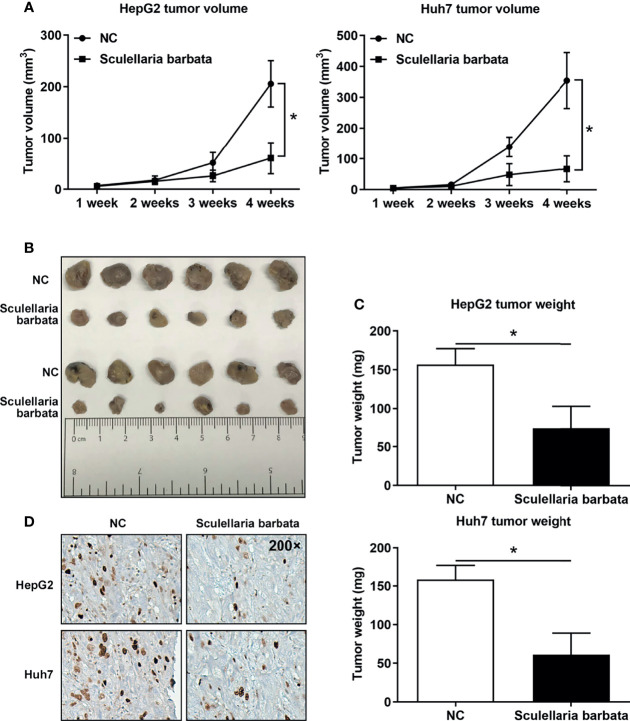
*Scutellaria barbata* inhibits HCC tumorigenicity *in vivo.* HCC cell lines (HepG2 and Huh7) were inoculated in nude mice separately, and subsequently, nude mice were treated by intragastric administration of *S. barbata* or PBS, and **(A)** volume, **(B)** size, and **(C)** weights of xenograft tumors were summarized. **(D)** IHC detected the expression of Ki-67 protein in the xenograft tumors. Magnification: 200×. *p < 0.05.

The above data suggested that *S. barbata* might be a valid choice to inhibit HCC tumorigenicity *in vivo*. Therefore, HCC cell lines (HepG2 and Huh7) were inoculated in nude mice separately, and subsequently, nude mice were treated by intragastric administration of *S. barbata*. The volume, size, and weight of the xenograft tumors in nude mice were measured. Results showed that most of the tumors without *S. barbata* treatment were significantly larger and heavier than those treated with *S. barbata* (**Figures 4A–C**). Furthermore, the expression of Ki-67 protein in the xenograft tumors were measured by IHC. Results showed that Ki-67 protein in without *S. barbata* treatment was significantly higher than those treated with *S. barbata* (**Figure 4D**). These data suggested that *S. barbata* suppressed HCC tumorigenicity *in vivo.*

### *Scutellaria barbata* Inhibits HCC Tumorigenicity by Inducing Ferroptosis of HCC Cells *In Vivo*

**Figure 5:**
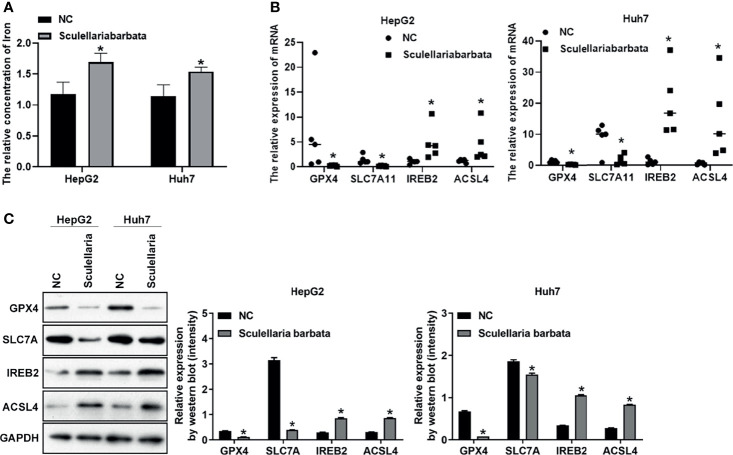
*Scutellaria barbata* inhibits HCC tumorigenicity by inducing ferroptosis of HCC cells *in vivo.* **(A)** Iron concentration in tumors detected by ELISA. **(B)** The level of mRNA in tumors detected by RT-PCR. **(C)** The level of protein in tumors detected by Western blot analysis. *p < 0.05.

To identify whether *S. barbata* inhibited HCC tumorigenicity by inducing ferroptosis *in vivo*, iron concentration in tumors was dramatically increased by *S. barbata* (**Figure 5A**). Moreover, both mRNA and protein levels of SLC7A11 in tumors were reduced after *S. barbata* treatment (**Figures 5B, C** **)**. These data together suggested that *S. barbata* inhibited HCC tumorigenicity *in vivo* by inducing ferroptosis of HCC cells.

## Discussion

Despite significant advances in clinical treatment of HCC in recent years, it remains one of the leading causes of cancer-related death worldwide. The difficulty in treatment and poor prognosis of patients with HCC lies in our limited choice of effective drugs for HCC, which suggests that we urgently need to develop new treatment methods for HCC. Ferroptosis is a new type of cell death characterized by accumulation of intracellular reactive oxygen species, which is iron dependent and non-apoptotic (34, 35). It is related to the occurrence and development of a variety of diseases, including liver cancer (32). TCM is a valuable medical method and resource in China, which has a significant effect on the treatment of HCC (36). However, there are very few studies on the regulation of ferroptosis in TCM, and only one study shows that artemisinin can promote ferroptosis in cancer cells (37). Therefore, this present study focused on the effect of TCM-inducing ferroptosis of HCC cells and provided theoretical basis for explaining the mechanism of TCM in treating HCC to find new drugs for treating HCC patients.

*Scutellaria barbata* has been wildly used in treatments for liver diseases. Several studies in human, cell, and animal have indicated the preventive effects of *S. barbata* on HCC. For example, a cohort study has revealed that *S. barbata* dramatically reduces HCC risk in patients with chronic hepatitis B, which is a major inducer of HCC. Moreover, total flavonoids of *S. barbata* prohibits HCC cell invasion through regulating TIMP expression (38). As the active component in *S. barbata*, pheophorbide a, C_35_H_36_N_4_O_5_ (molecular weight, 593), attenuates multidrug resistance of HCC cell line (39). In animal experiments, *S. barbata* polysaccharides suppress HCC cell growth in the H22 hepatoma−bearing Kunming mice (40). Furthermore, *S. barbata* crude extract has a protective effect against liver tumorigenesis in rat. However, the role of *S. barbata* on ferroptosis is not clear. Results of our study indicated that *S. barbata* induced ferroptosis of HCC cells, suggesting that *S. barbata* may prevent HCC development through ferroptosis.

Recent studies have identified the genes and pathways related to ferroptosis in HCC. Therefore, we first screened the target genes and pathways of *Scutellaria* in HCC to clarify the related mechanism. We found out that *S. barbata* decreased significantly the mRNA and protein expression levels of anti-ferroptosis in lipid ROS metabolism-related genes, namely, GPX4 and SLC7A11; meanwhile, *S. barbata* increased significantly the mRNA expression levels of iron perioxidation-related gene IREB2 and CoA metabolism-related gene ACSL4, which both are ferroptosis-positive regulators. We came to the conclusion that *S. barbata* induced ferroptosis of HCC cells by promoting iron perioxidation and lipid ROS metabolism. In the end, we constructed an animal model of HCC through tumor formation in nude mice and found out that *S. barbata* significantly inhibited the growth of tumor cells in nude mice. Furthermore, *S. barbata* could significantly decrease the expression level of GPX4 and SLC7A11 and increase the expression level of IREB2 and ACSL4 in nude mice, which enabled the ability of *S. barbata* to increase iron concentration in nude mice. Specific inhibitors of ferroptosis have been shown to alleviate organ damage in several clinical models, including a model of heart disease. Fang et al. found that mice have altered cardiac iron homeostasis and develop mild cardiomyopathy with aging, which is rescued by treating the mice with the ferroptosis inhibitor ferrostatin-1 (41). Sorafenib, against advanced HCC, also exerts cytotoxic effects *via* the induction of ferroptosis (42). Next, we will explore the role of ferrostatin-1. *Scutellaria barbata* inhibits hepatocellular carcinoma tumorigenicity by inducing ferroptosis of hepatocellular carcinoma cells, which may be rescued by ferroptosis inhibitor.

In summary, this present study demonstrated *S. barbata* induced HCC cells ferroptosis by promoting HCC cells’ iron perioxidation and lipid ROS metabolism. In addition, we also found out that *S. barbata* inhibited HCC tumorigenesis *in vivo* by inducing ferroptosis of HCC cells. We provided theoretical basis for explaining the mechanism of a kind of TCM in treating HCC and offered therapeutic opportunities for a wide range of HCC patients.

## Data Availability Statement

The original contributions presented in the study are included in the article/supplementary material. Further inquiries can be directed to the corresponding authors.

## Ethics Statement

The animal study was reviewed and approved by Ethic Committee of The Third Affiliated Hospital of Sun Yat-Sen University.

## Author Contributions

MD and Y-W L conceived and directed this research and contributed to the project design. YL and JZ conducted the experiments and analyzed the data. KZ, YC, WW, and HC helped provide data and comments on the manuscript. Reagents, materials, and contributions were contributed by ZZ. YL and JZ were responsible for drafting the manuscript. All authors contributed to the article and approved the submitted version.

## Conflict of Interest

The authors declare that the research was conducted in the absence of any commercial or financial relationships that could be construed as a potential conflict of interest.

## Publisher’s Note

All claims expressed in this article are solely those of the authors and do not necessarily represent those of their affiliated organizations, or those of the publisher, the editors and the reviewers. Any product that may be evaluated in this article, or claim that may be made by its manufacturer, is not guaranteed or endorsed by the publisher.

## References

[1] Forner A Llovet JM Bruix J. Hepatocellular Carcinoma. Lancet (2012) 379:1245–55. doi: 10.1016/s0140-6736(11)61347-0 PMC13537732 22353262

[2] Ghouri YA Mian I Rowe JH. Review of Hepatocellular Carcinoma: Epidemiology, Etiology, and Carcinogenesis. J Carcinog (2017) 16:1. doi: 10.4103/jcar.JCar_9_16 28694740 PMC5490340

[3] Zhou M Wang H Zhu J Chen W Wang L Liu S. Cause-Specific Mortality for 240 Causes in China During 1990-2013: A Systematic Subnational Analysis for the Global Burden of Disease Study 2013. Lancet (2016) 387:251–72. doi: 10.1016/s0140-6736(15)00551-6 26510778

[4] Kew MC. Epidemiology of Hepatocellular Carcinoma in Sub-Saharan Africa. Ann Hepatol (2013) 12:173–82. doi: 10.1016/S1665-2681(19)31354-7 23396727

[5] Altekruse SF McGlynn KA Dickie LA Kleiner DE. Hepatocellular Carcinoma Confirmation, Treatment, and Survival in Surveillance, Epidemiology, and End Results Registries, 1992-2008. Hepatology (2012) 55:476–82. doi: 10.1002/hep.24710 PMC3868012 21953588

[6] Wu MC. Traditional Chinese Medicine in Prevention and Treatment of Liver Cancer: Function, Status and Existed Problems. Zhong Xi Yi Jie He Xue Bao (2003) 1:163–4. doi: 10.3736/jcim20030302 15339547

[7] Gong B Kao Y Zhang C Sun F Zhao H. Systematic Investigation of Scutellariae Barbatae Herba for Treating Hepatocellular Carcinoma Based on Network Pharmacology. Evid Based Complement Alternat Med (2018) 2018:4365739. doi: 10.1155/2018/4365739 30584453 PMC6280310

[8] Wang Y Ma J Chow SC Li CH Xiao Z Feng R. A Potential Antitumor Ellagitannin, Davidiin, Inhibited Hepatocellular Tumor Growth by Targeting EZH2. Tumour Biol (2014) 35:205–12. doi: 10.1007/s13277-013-1025-3 23897557

[9] Tao X Wang P Yang X Yao H Liu J Cao Y. Inhibitory Effect of Ardipusilloside-I on Lewis Pulmonary Carcinoma and Hepatocarcinoma SMMC-7721. Zhong Yao Cai (2005) 28:574–7. 16252725

[10] Chen Y Xu SS Chen JW Wang Y Xu HQ Fan NB. Anti-Tumor Activity of Annona Squamosa Seeds Extract Containing Annonaceous Acetogenin Compounds. J Ethnopharmacol (2012) 142:462–6. doi: 10.1016/j.jep.2012.05.019 22609808

[11] Huang J Tang XH Ikejima T Sun XJ Wang XB Xi RG. A New Triterpenoid From Panax Ginseng Exhibits Cytotoxicity Through P53 and the Caspase Signaling Pathway in the Hepg2 Cell Line. Arch Pharm Res (2008) 31:323–9. doi: 10.1007/s12272-001-1159-8 18409045

[12] Li X Guan YS Zhou XP Sun L Liu Y He Q. Anticarcinogenic Effect of 20(R)-Ginsenoside Rg3 on Induced Hepatocellular Carcinoma in Rats. Sichuan Da Xue Xue Bao Yi Xue Ban (2005) 36:217–20. 15807271

[13] Chen JC Chung JG Chen LD. Gypenoside Induces Apoptosis in Human Hep3B and HA22T Tumour Cells. Cytobios (1999) 100:37–48. 10643643

[14] Hu B Wang SS Du Q. Traditional Chinese Medicine for Prevention and Treatment of Hepatocarcinoma: From Bench to Bedside. World J Hepatol (2015) 7:1209–32. doi: 10.4254/wjh.v7.i9.1209 PMC4438495 26019736

[15] Liu H Chou GX Wang JM Ji LL Wang ZT. Steroidal Saponins From the Rhizomes of Dioscorea Bulbifera and Their Cytotoxic Activity. Planta Med (2011) 77:845–8. doi: 10.1055/s-0030-1250633 21165820

[16] Xie RF Li ZC Gao B Shi ZN Zhou X. Bufothionine, a Possible Effective Component in Cinobufocini Injection for Hepatocellular Carcinoma. J Ethnopharmacol (2012) 141:692–700. doi: 10.1016/j.jep.2011.12.018 22210051

[17] Gong K Chen C Zhan Y Chen Y Huang Z Li W. Autophagy-Related Gene 7 (ATG7) and Reactive Oxygen Species/Extracellular Signal-Regulated Kinase Regulate Tetrandrine-Induced Autophagy in Human Hepatocellular Carcinoma. J Biol Chem (2012) 287:35576–88. doi: 10.1074/jbc.M112.370585 PMC3471698 22927446

[18] Chang UM Li CH Lin LI Huang CP Kan LS Lin SB. A Ganoderma Triterpene, Induces Senescence in Hepatoma Hepg2 Cells. Life Sci (2006) 79:1129–39. doi: 10.1016/j.lfs.2006.03.027 16635496

[19] Do Van B Gouel F Jonneaux A Timmerman K Gelé P Pétrault M. Ferroptosis, a Newly Characterized Form of Cell Death in Parkinson’s Disease That is Regulated by PKC. Neurobiol Dis (2016) 94:169–78. doi: 10.1016/j.nbd.2016.05.011 27189756

[20] Eling N Reuter L Hazin J Hamacher-Brady A Brady NR. Identification of Artesunate as a Specific Activator of Ferroptosis in Pancreatic Cancer Cells. Oncoscience (2015) 2:517–32. doi: 10.18632/oncoscience.160 PMC4468338 26097885

[21] Firestone RA. Low-Density Lipoprotein as a Vehicle for Targeting Antitumor Compounds to Cancer Cells. Bioconjug Chem (1994) 5:105–13. doi: 10.1021/bc00026a002 8031872

[22] Friedmann Angeli JP Schneider M Proneth B Tyurina YY Tyurin VA Hammond VJ. Inactivation of the Ferroptosis Regulator Gpx4 Triggers Acute Renal Failure in Mice. Nat Cell Biol (2014) 16:1180–91. doi: 10.1038/ncb3064 PMC4894846 25402683

[23] Galmiche A Chauffert B Barbare JC. New Biological Perspectives for the Improvement of the Efficacy of Sorafenib in Hepatocellular Carcinoma. Cancer Lett (2014) 346:159–62. doi: 10.1016/j.canlet.2013.12.028 24380851

[24] Guo J Xu B Han Q Zhou H Xia Y Gong C. Ferroptosis: A Novel Anti-Tumor Action for Cisplatin. Cancer Res Treat (2018) 50:445–60. doi: 10.4143/crt.2016.572 PMC5912137 28494534

[25] Hao S Yu J He W Huang Q Zhao Y Liang B. Cysteine Dioxygenase 1 Mediates Erastin-Induced Ferroptosis in Human Gastric Cancer Cells. Neoplasia (2017) 19:1022–32. doi: 10.1016/j.neo.2017.10.005 PMC5686465 29144989

[26] Harrison PM Arosio P. The Ferritins: Molecular Properties, Iron Storage Function and Cellular Regulation. Biochim Biophys Acta (1996) 1275:161–203. doi: 10.1016/0005-2728(96)00022-9 8695634

[27] Hayano M Yang WS Corn CK Pagano NC Stockwell BR. Loss of Cysteinyl-Trna Synthetase (CARS) Induces the Transsulfuration Pathway and Inhibits Ferroptosis Induced by Cystine Deprivation. Cell Death Differ (2016) 23:270–8. doi: 10.1038/cdd.2015.93 PMC4716307 26184909

[28] Dixon SJ Lemberg KM Lamprecht MR Skouta R Zaitsev EM Gleason CE. Ferroptosis: An Iron-Dependent Form of Nonapoptotic Cell Death. Cell (2012) 149:1060–72. doi: 10.1016/j.cell.2012.03.042 PMC3367386 22632970

[29] Ou W Mulik RS Anwar A McDonald JG He X Corbin IR. Low-Density Lipoprotein Docosahexaenoic Acid Nanoparticles Induce Ferroptotic Cell Death in Hepatocellular Carcinoma. Free Radic Biol Med (2017) 112:597–607. doi: 10.1016/j.freeradbiomed.2017.09.002 28893626 PMC5848495

[30] Bersuker K Hendricks JM Li Z Magtanong L Ford B Tang PH. The Coq Oxidoreductase FSP1 Acts Parallel to GPX4 to Inhibit Ferroptosis. Nature (2019) 575:688–92. doi: 10.1038/s41586-019-1705-2 PMC6883167 31634900

[31] Liu T Jiang L Tavana O Gu W. The Deubiquitylase OTUB1 Mediates Ferroptosis *via* Stabilization of SLC7A11. Cancer Res (2019) 79:1913–24. doi: 10.1158/0008-5472.can-18-3037 PMC6467774 30709928

[32] Nie J Lin B Zhou M Wu L Zheng T. Role of Ferroptosis in Hepatocellular Carcinoma. J Cancer Res Clin Oncol (2018) 144:2329–37. doi: 10.1007/s00432-018-2740-3 PMC11813439 30167889

[33] Doll S Proneth B Tyurina YY Panzilius E Kobayashi S Ingold I. ACSL4 Dictates Ferroptosis Sensitivity by Shaping Cellular Lipid Composition. Nat Chem Biol (2017) 13:91–8. doi: 10.1038/nchembio.2239 PMC5610546 27842070

[34] Yu H Guo P Xie X Wang Y Chen G. Ferroptosis, a New Form of Cell Death, and its Relationships With Tumourous Diseases. J Cell Mol Med (2017) 21:648–57. doi: 10.1111/jcmm.13008 PMC5345622 27860262

[35] Stockwell BR Friedmann Angeli JP Bayir H Bush AI Conrad M Dixon SJ. Ferroptosis: A Regulated Cell Death Nexus Linking Metabolism, Redox Biology, and Disease. Cell (2017) 171:273–85. doi: 10.1016/j.cell.2017.09.021 PMC5685180 28985560

[36] Hu B An HM Wang SS Chen JJ Xu L. Preventive and Therapeutic Effects of Chinese Herbal Compounds Against Hepatocellular Carcinoma. Molecules (2016) 21:142. doi: 10.3390/molecules21020142 26828466 PMC6274246

[37] Chen GQ Benthani FA Wu J Liang D Bian ZX Jiang X. Artemisinin Compounds Sensitize Cancer Cells to Ferroptosis by Regulating Iron Homeostasis. Cell Death Differ (2020) 27:242–54. doi: 10.1038/s41418-019-0352-3 PMC7205875 31114026

[38] Dai ZJ Wang BF Lu WF Wang ZD Ma XB Min WL. Total Flavonoids of Scutellaria Barbata Inhibit Invasion of Hepatocarcinoma *via* MMP/TIMP *In Vitro* . Molecules (2013) 18:934–50. doi: 10.3390/molecules18010934 PMC6269956 23344202

[39] Tang PM Chan JY Zhang DM Au SW Fong WP Kong SK. Pheophorbide a, an Active Component in Scutellaria Barbata, Reverses P-Glycoprotein-Mediated Multidrug Resistance on a Human Hepatoma Cell Line R-Hepg2. Cancer Biol Ther (2007) 6:504–9. doi: 10.4161/cbt.6.4.3814 17457045

[40] Li L Xu X Wu L Zhu H He Z Zhang B. Scutellaria Barbata Polysaccharides Inhibit Tumor Growth and Affect the Serum Proteomic Profiling of Hepatoma H22−Bearing Mice. Mol Med Rep (2019) 19:2254–62. doi: 10.3892/mmr.2019.9862 PMC6390040 30664217

[41] Fang X Cai Z Wang H Han D Cheng Q Zhang P. Loss of Cardiac Ferritin H Facilitates Cardiomyopathy *via* Slc7a11-Mediated Ferroptosis. Circ Res (2020) 127:486–501. doi: 10.1161/circresaha.120.316509 32349646

[42] Liu Y Zhang X Zhang J Tan J Li J Song Z. Development and Validation of a Combined Ferroptosis and Immune Prognostic Classifier for Hepatocellular Carcinoma. Front Cell Dev Biol (2020) 8:596679:596679. doi: 10.3389/fcell.2020.596679 33425905 PMC7785857

